# Low carbohydrate and psychoeducational programs show promise for the treatment of ultra-processed food addiction

**DOI:** 10.3389/fpsyt.2022.1005523

**Published:** 2022-09-28

**Authors:** Jen Unwin, Christine Delon, Heidi Giæver, Clarissa Kennedy, Molly Painschab, Frida Sandin, Charlotte Schön Poulsen, David A. Wiss

**Affiliations:** ^1^Public Health Collaboration, London, United Kingdom; ^2^Sweet Sobriety, Belgrade, MT, United States; ^3^Sweet Sobriety, Parry Sound, ON, Canada; ^4^Levasockerfri, Linkoping, Sweden; ^5^Nutrition in Recovery LLC, Los Angeles, CA, United States

**Keywords:** addiction, sugar, processed-food, low-carbohydrate diet, ketogenic diets

## Abstract

Food addiction, specifically ultra-processed food addiction, has been discussed in thousands of peer-reviewed publications. Although 20% of adults meet criteria for this condition, food addiction is not a recognized clinical diagnosis, leading to a dearth of tested treatment protocols and published outcome data. Growing numbers of clinicians are offering services to individuals on the basis that the food addiction construct has clinical utility. This audit reports on clinical teams across three locations offering a common approach to programs delivered online. Each team focused on a whole food low-carbohydrate approach along with delivering educational materials and psychosocial support relating to food addiction recovery. The programs involved weekly sessions for 10–14 weeks, followed by monthly support. The data comprised pre- and post- program outcomes relating to food addiction symptoms measured by the modified Yale Food Addiction Scale 2.0, ICD-10 symptoms of food related substance use disorder (CRAVED), mental wellbeing as measured by the short version of the Warwick Edinburgh Mental Wellbeing Scale, and body weight. Sample size across programs was 103 participants. Food addiction symptoms were significantly reduced across settings; mYFAS2 score −1.52 (95% CI: −2.22, −0.81), CRAVED score −1.53 (95% CI: −1.93, −1.13) and body weight was reduced −2.34 kg (95% CI: −4.02, −0.66). Mental wellbeing showed significant improvements across all settings; short version Warwick Edinburgh Mental Wellbeing Scale 2.37 (95% CI: 1.55, 3.19). Follow-up data will be published in due course. Further research is needed to evaluate and compare long-term interventions for this complex and increasingly burdensome biopsychosocial condition.

## Graphical Abstract

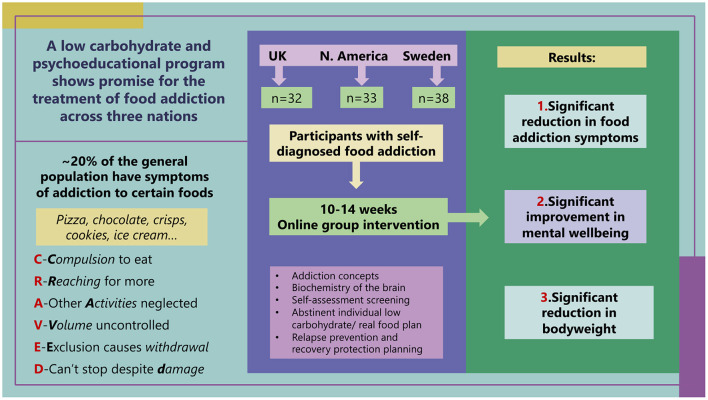

## Introduction

Food addiction (FA) was first described in 1956 (1). Considerable debate has continued and it remains unresolved if FA is a distinct disorder warranting official recognition (2–4). To date, FA has not been classified in the Diagnostic and Statistical Manual of Mental Disorders (5) (DSM-5) or in the International Classification of Diseases (6) (ICD-10). There is also ongoing discussion amongst clinicians as to how to refer to this disorder. For the purposes of this paper, we will use the term food addiction to refer to dependency behaviors relating to sugar and processed foods, although it is increasingly being referred to as ultra-processed food addiction (7).

FA is operationalized using the Yale Food Addiction Scale (YFAS) originally published in 2009 (8) and more recently the YFAS 2.0 (9). In 2015, Schulte et al. reported pizza, chocolate, chips (crisps), cookies (biscuits) and ice cream as the five most problematic foods for those with FA symptoms (10). In recent years, increasing numbers of articles have described FA symptoms (11), prevalence (12), and possible mechanisms (13, 14) in both animals and diverse human populations worldwide. The most recent estimates suggest that the worldwide prevalence of FA is ~20% and that it positively correlates with BMI and eating disorders (15).

The symptoms of FA are captured using the 11 criteria for substance use disorder (SUD) from the DSM-5 (5) and applying those to foods high in refined carbohydrates/sugar, fat, and salt. Two or three symptoms indicate mild SUD, four or five is moderate and six or more indicates severe SUD. The criteria include:

- Consuming the substance in larger amounts or for longer than intended.
- Efforts to cut down or stop using the substance but not managing to.
- Time spent getting, using, or recovering from the substance.
- Cravings and urges to use the substance.
- Not managing to perform at work, home or school because of substance use.
- Continuing to use the substance despite causing problems in relationships.
- Giving up important social, occupational, or leisure activities because of substance use.
- Using the substance repeatedly despite harmful consequences.
- Continuing to use the substance despite physical or psychological problem caused or worsened by the substance.
- Needing more of the substance to get the desired effect.
- Development of withdrawal symptoms which are relieved by consumption of substance.

Similarly, there are six criteria from the ICD-10 (6), where three or more symptoms indicate SUD:

- “Craving,” a strong desire or urge to use the substance.
- Difficulty controlling the onset, duration, amount, and termination of substance use.
- Increasing priority of substance use over other activities over time.
- Increased tolerance and the need to increase consumption over time.
- Physiological features of withdrawal when trying to abstain.
- Continued use of the substance despite mental or physical harm.

Clinicians who work with persons with type 2 diabetes, obesity, and metabolic syndrome will likely recognize these behaviors in their patients, particularly those who struggle to follow nutrition and lifestyle advice consistently. It has been shown that an understanding of addiction-like eating behavior can shift the blame narrative away from assumptions of “personal responsibility” and thereby reduce stigma associated with eating behavior (16, 17).

Prevalence estimates of FA are consistently highest in clinical samples of eating disorders (EDs), which has led some authors to urge for ED screening and careful assessment before determining proper diagnosis and treatment (18). Specifically, individuals with bulimia nervosa (BN) have the highest prevalence of FA (48–95%), followed by binge eating disorder (BED; 55–80%), and then anorexia nervosa (AN; 44–70%) (15, 18–20). It has been suggested that efforts to restrain eating, engage in compensatory behaviors (e.g., purging), or maintain body weights below normal might lead to increases in self-reported FA scores (7). Meanwhile, it can be established that FA symptoms exist independently of ED symptoms, thus it can be conceptualized as a distinct disorder warranting targeted interventions (7, 18). More research is needed in this area.

Several neurobiological mechanisms have been proposed to explain FA. Wiss et al. stated that “evidence is accumulating on the overlap of neural circuitry and commonalities between drug abuse and FA in humans” (13). These authors propose that FA in humans is similar to nicotine or caffeine addiction and that hyperpalatable foods can “hijack” reward centers in the brain, impairing decision-making processes in ways that can be subtle or quite obvious to the person (and those close to them). Similarly, Lindgren et al. found support for the concept of FA *via* overlapping neural mechanisms with drug and alcohol addiction: a dampening of dopamine signaling and downregulation of the μ-opioid receptor, “coupled with impairment of prefrontal regions that are involved in inhibitory control” (14). The authors add that further research is needed on the complex interaction between these processes and the hormones that modulate feeding behavior. Their discussion points to the challenge of designing interventions for FA because unlike other SUDs, total abstinence from food is not an available option.

A range of possible interventions for FA symptoms have been proposed including medications (21), cognitive behavioral therapy (22), brain stimulation (23), psychoeducation (24), bariatric surgery (25), low-calorie diets (26), probiotics (27), and “infra slow” brain training (28). No data have been presented for medication (21), cognitive behavioral therapy (22) or brain stimulation (23). Eleven obese women reported reduced cravings after infra slow brain training, however there was no follow up (28). A 6-week uncontrolled psychoeducational program for 66 women with BN showed FA severity can improve but still found 73% FA post intervention (24). A study of 44 people undergoing bariatric surgery showed a reduction of 32–2% with food addiction symptoms at 6 months (25). A low-calorie diet in 11 people with obesity and FA was found to normalize brain activation compared to people with obesity without FA. However, follow up was only 3 months and no details of the diet were given (26). In a randomized trial of probiotics for women with obesity and FA, the active treatment led to greater improvements in oxytocin levels and eating behavior, however there was no follow up.

Several authors affirm that low-carbohydrate approaches have therapeutic potential for treating FA symptoms (29). They propose that ultra-processed, refined, or high-glycemic index carbohydrates are a possible “trigger” mediating neurochemical response that is similar to that seen in addictions. The carbohydrate-insulin model of obesity supports observations of these foods triggering abnormal blood sugar and insulin spikes subsequently leading to changes in metabolic and neurobiological signaling (30). Carmen et al. published a case series of three patients with obesity, BED, and FA managed over 6–7 months on a low-carbohydrate ketogenic approach with no adverse effects (31). They were followed up over 9–17 months. Both binge eating and FA symptoms improved, accompanied by a 10–24% body weight loss. Interventions for FA must be able to demonstrate sustainable changes to symptoms and mental wellbeing. FA recovery can be achieved without overemphasis on weight which can detract from the clinical utility of the construct as a behavioral disorder (7).

In a recent poll of an online food addiction professional group, we found that 20 out 25 practitioners recommend low-carbohydrate or ketogenic food plans as part of their interventions (unpublished data). Although this proportion is subject to selection bias, it clarifies that carbohydrate restriction is a common clinical practice for the treatment of FA. Other practitioners include grains and fruit in their plans. No previous audits of practice outcomes in food addiction have been published to our knowledge. The current audit describes the pre- and post-intervention data from practices in three different countries offering online group interventions for people self-identifying as having FA, including an “abstinent” low-carbohydrate “real food” approach and biopsychosocial education focused on addiction and recovery.

## Materials and methods

Clinics in three locations [the United Kingdom (UK); North America (NA); Sweden (SE)] already offering similar online programs for people with FA used the same measures for screening and follow up. The ethics protocol for the National Health Service in the UK was reviewed and indicated that since the project was an audit of pre-existing routine practice and participants were self-referred, formal ethical review was not required.

### Participants

Participants in the programs typically made contact *via* social media and mailing list advertisements by the authors. Participants were screened through online interviews by the appropriate clinician to confirm self-identified FA symptoms. None of the programs accepted people under 18 years of age, pregnant, having serious mental health problems requiring ongoing specialist psychiatric support, or any doctor requesting exclusion. Each participant was given information about the program and audit and the opportunity to ask questions. Participants completed a consent form as part of the initial data collection to affirm that their anonymized data could be used in the audit of the programs. Participants' data were identified by a unique code to ensure anonymity. An information sheet (UK) and protocols are included as Supplementary materials. Participants paid a reduced fee (NA, SE) or donation (UK) to participate.

### Power calculation

Data collection points were scheduled before and after the online group and at 6 months, 1 year, 18 months and 2 year follow up. The current paper audits the data available to date, which is the initial pre- and post- active intervention data as of June 2022. Power calculations using the main outcome measures of the mYFAS2 (32) and the short version of the Warwick Edinburgh Mental Wellbeing Scale (33) (SWEMWBS) indicated that 26 participants were needed to complete the 2-year follow-up in each location, for a total of 78 total participants. Each location aimed to have 60–70 participants complete baseline data to ensure adequate numbers at 2-year follow-up. The total sample size at the time of this audit is *n* = 103 (UK *n* = 32; NA *n* = 33; SE *n* = 38).

### Measures

The mYFAS2 is a short version of the YFAS 2.0 (34). The mYFAS2 includes 13 items: one item for each of the 11 FA criteria in the DSM-5 for SUD and two items for the assessment of clinically significant impairment or distress. One example item is: I ate until I was physically ill. There are eight frequency choices from never to every day. The mYFAS2 has good reliability and convergent and discriminant validity (34). The scale can be scored as total number of criteria met (0–11, reported here) or as an indication of a clinical diagnosis and severity.

A brief screening tool for FA symptoms based on the six ICD-10 criteria for SUD (6) was developed by HG and JU as a simple tool for clinicians. CRAVED, which has not been formally validated, is described and included in the Supplementary materials. Participants were asked to rate whether they had experienced the symptom in the last month (yes or no, possible score 0–6). An example item is: I had such a strong desire or sense of compulsion at the thought of eating these foods, that I could not resist the urge to eat them. A score of 3 or more out of six indicates a potential SUD according to ICD-10 (6).

The SWEMWBS is a short version of the Warwick-Edinburgh Mental Wellbeing Scale (34). The scale was developed to monitor mental wellbeing in the general population and for the evaluation of programs designed to improve mental wellbeing. There are seven statements relating to functioning such as I've been thinking clearly with five response categories from none of the time to all of the time. The measure has good construct and external validity and test-retest reliability (34). Scores range from 7 to 35, higher scores indicating more positive wellbeing. The England population mean is 23.6 (34).

The following data were also collected: age, gender, and weight (kg). The online survey took ~10 min to complete.

### Programs

The programs consisted of 10–14 weeks of 90–120-min sessions in groups of 11–40 participants. The variation is due to each location having their own set of program materials and methods. Sessions consisted of educational content delivered live or pre-recorded, coaching discussions, and assigned reflections. The content of the programs included: understanding addiction concepts and biochemistry, self-assessment screening and reflection, abstinent low-carbohydrate individualized “real food” plan, imagining life beyond FA, new habits and tastes, resilience, relapse prevention planning, and personal lifestyle planning. A comparison of the three group programs and an example food plan (UK) are included in the Supplementary materials. Abstinence from sugar, grains, processed food and any foods the individual participants were unable to moderate (e.g., peanut butter) was emphasized. Following the active program phase, participants joined a monthly 60-min facilitated online support group, which will continue for 2 years. All groups also established independently their own support group chats and online meetings.

### Data collection and analysis

Participants entered their data into online forms which were analyzed using R v4.0.2. *P*-values were calculated using the Wilcoxon rank sum test with continuity correction, and a value below 0.05 was considered statistically significant. Summary statistics were calculated using random effects models and the DerSimonian-Laird estimate (35) and visualized as forest plots using the meta package, version 4.13-0 and the metamean function.

## Results

**Figure 1:**
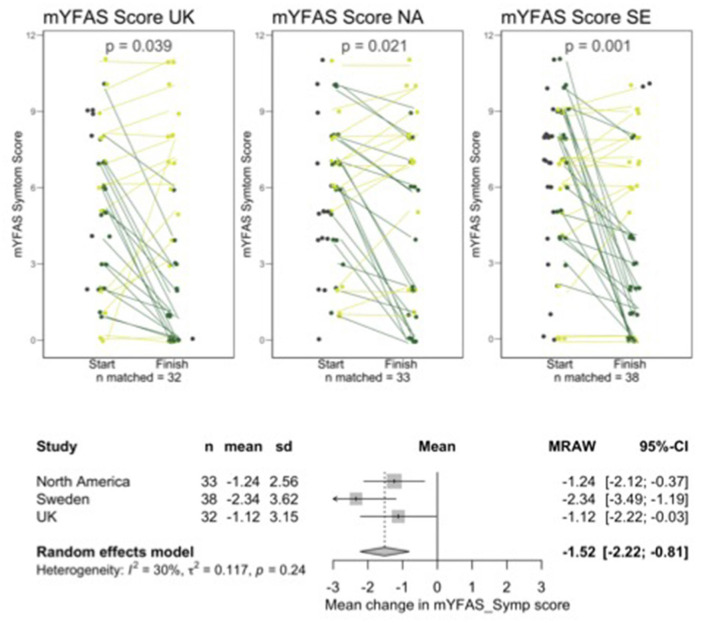
Line and forest plots for mYFAS2 symptom score. Dark green indicates improved scores, light green indicates worsening score or no change. Dark gray data points without a line represent people who started but did not finish or who completed a follow up questionnaire but could not be matched with a starting questionnaire.

Not all participants were available for follow-up and a small number of participants who completed follow-up data could not be matched to baseline data due to them entering unidentifiable codes. There were 32, 33, and 38 sets of matched data for UK, NA, and SE, respectively. Graphs shown in Figure 1 through Figure 4 show all available data points for pre -and post-intervention data, including participants who were not available to follow up and unmatched participants but all analyses of the change from pre- to post-intervention were carried out on the matched pairs of data. Table 1 shows retention data to date.

**Table 1:** Data recruitment and retention.

	**UK**	**NA**	**SE**
Expressed an interest	49	136	138
Screened	41	115	98
Accepted	40	82	83
Baseline data	40	71	60
Average group size	13.3	26.6	30
Sessions completed	33	34	42
Post-intervention data available	33	33	40
Matched pairs for analysis	32	33	38

The mean age of UK participants was 50 years (SD = 12), in NA 49 (SD = 12) and in SE 47 (SD = 9.8). Participants were predominantly female (91% UK, 97% NA, and 100% SE).

Table 2 summarizes the UK data. Decrease in mYFAS2 scores was significant (mean reduction 1.1, SD 3.1, *p* = 0.039). Reduction in CRAVED was significant (mean reduction 1.7, SD 2.1, *p* < 0.001), as was increases in SWEMWBS (mean increase 3.1, SD 3.2, *p* < 0.001). Reduction in weight was also significant (Mean loss 2.5 kg, SD 6.5, *p* = 0.02).

**Table 2:** Summary data for UK participants.

**Value**	***N***	**Median**	**Mean**	***P*** **-value**
		**Q1Q3**	**SD**	**for**
				**pre-post-**
Age (years)	32	50 (41, 60)	50 (12)	
Height (cm)	32	165 (160, 170)	165 (6.9)	
Weight pre (kg)	32	88 (72, 99)	88 (19)	
Weight post	32	82 (71, 95)	85 (20)	0.022^*^
Weight loss	32	2.5 (-1.2, 5.5)	2.8 (6.5)	
mYFAS2 symp pre	32	5.0 (2.0, 7.0)	4.9 (3.2)	
mYFAS2 symp post	32	3.0 (0.0, 7.0)	3.8 (3.7)	0.039^*^
mYFAS2 symp loss	32	1.0 (-1.0, 3.0)	1.1 (3.1)	
CRAVED pre	32	5.0 (4.0, 6.0)	4.9 (1.1)	
CRAVED post	32	3.5 (1.8, 5.0)	3.2 (2.0)	<0.001^***^
CRAVED loss	32	2.0 (0.0, 3.0)	1.7 (2.1)	
SWEMWBS pre	32	20 (19, 21)	20 (2.9)	
SWEMWBS post	32	23 (21, 25)	23 (4.6)	<0.001^***^
SWEMWBS loss	32	−3.1 (−4.9, −1.4)	−3.1 (3.2)	

* *P* < 0.05;

**P < 0.01;

and

*** *P* <0.001.

Table 3 summarizes the NA data. Reduction in mYFAS2 scores was significant (mean reduction 1.2, SD 2.6, *p* = 0.021). Reduction in CRAVED was significant (mean reduction 1.8, SD 2.2, *p* < 0.001), as were increases in SWEMWBS (mean increase 1.6, SD 3.2, *p* = 0.008). Reduction in weight was also significant (mean loss 4.4 kg, SD 9.4, *p* = 0.001).

**Table 3:** Summary data for NA participants.

**Value**	***N***	**Median**	**Mean**	***P*** **-value**
		**Q1Q3**	**SD**	**for**
				**pre-post-**
Age (years)	33	48 (42, 58)	49 (12)	
Height (cm)	32	165 (159, 170)	165 (9.2)	
Weight pre (kg)	31	88 (71, 106)	91 (29)	
Weight post	32	81 (67, 96)	85 (28)	0.001^**^
Weight loss	30	2.5 (0.0, 5.6)	4.4 (9.4)	
mYFAS2 symp pre	33	6.0 (4.0, 8.0)	6.0 (2.8)	
mYFAS2 symp post	33	6.0 (1.0, 8.0)	4.8 (3.6)	0.021^*^
mYFAS2 symp loss	33	1.0 (−1.0, 2.0)	1.2 (2.6)	
CRAVED pre	33	5.0 (4.0, 6.0)	4.8 (1.2)	
CRAVED post	33	3.0 (1.0, 5.0)	3.0 (2.1)	<0.001^***^
CRAVED loss	33	1.0 (0.0, 4.0)	1.8 (2.2)	
SWEMWBS pre	33	22 (19, 23)	22 (3.0)	
SWEMWBS post	33	22 (21, 25)	23 (3.8)	0.008^**^
SWEMWBS loss	33	−0.9 (-2.4, 0.8)	−1.6 (3.2)	

* *P* < 0.05;

** *P* < 0.01;

and

*** *P* <0.001.

Table 4 summarizes the SE data. Reduction in mYFAS2 scores was significant (mean reduction 2.3, SD 3.6, *p* = 0.001). Reduction in CRAVED was significant (mean reduction 0.2, SD 1.2, *p* < 0.001), as was increases in SWEMWBS (mean increase 2.4, SD 3.3, *p* < 0.001). Reduction in weight was also significant (mean reduction 1.2 kg, SD 4.7, *p* = 0.01).

**Table 4:** Summary data for SE participants.

**Value**	***N***	**Median**	**Mean**	***P*** **-value**
		**Q1Q3**	**SD**	**for**
				**pre-post-**
Age (years)	38	46 (40, 56)	47 (9.8)	
Height (cm)	38	169 (163, 175)	169 (9.1)	
Weight pre (kg)	38	84 (75, 100)	87 (18)	
Weight post	37	83 (70, 97)	85 (19)	0.01^*^
Weight loss	37	1.3 (0.0, 4.0)	1.2 (4.7)	
mYFAS2 symp pre	38	7.0 (4.2, 9.0)	6.3 (2.9)	
mYFAS2 symp post	38	3.5 (1.0, 7.0)	4.0 (3.4)	0.001^**^
mYFAS2 symp loss	38	1.0 (0.0, 5.8)	2.3 (3.6)	
CRAVED pre	38	5.0 (4.2, 6.0)	5.0 (1.1)	
CRAVED post	38	4.0 (2.2, 5.8)	3.8 (2.0)	<0.001^***^
CRAVED loss	21	0.0 (0.0, 1.0)	0.2 (1.2)	
SWEMWB pre	38	20 (19, 23)	21 (2.7)	
SWEMWB post	38	23 (21, 25)	23 (3.3)	<0.001^***^
SWEMWB loss	38	−1.7 (−4.1, 0.0)	−2.4 (3.3)	

* P < 0.05;

** P < 0.01;

and

*** P <0.001.

**Figure 2:**
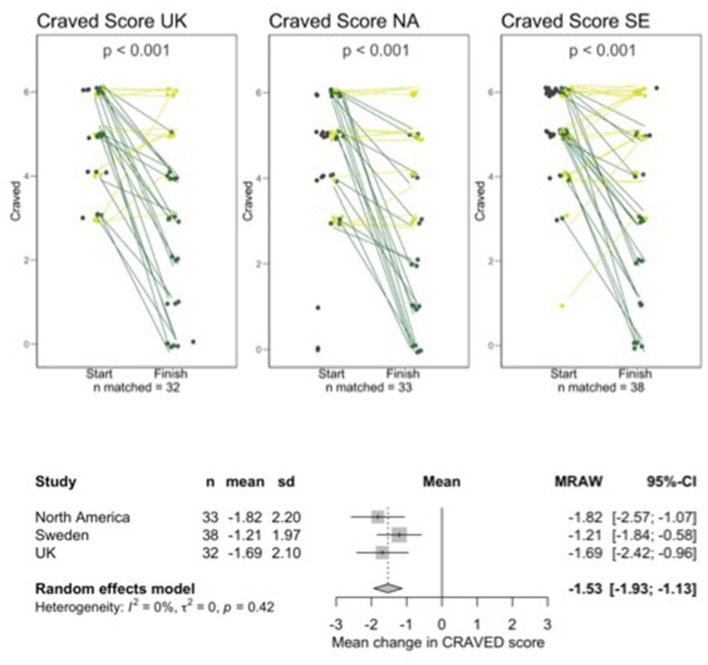
Line and forest plots for CRAVED. Dark green indicates improved scores, light green indicates worsening score or no change. Dark gray data points without a line represent people who started but did not finish or who completed a follow up questionnaire but could not be matched with a starting questionnaire.

**Figure 3:**
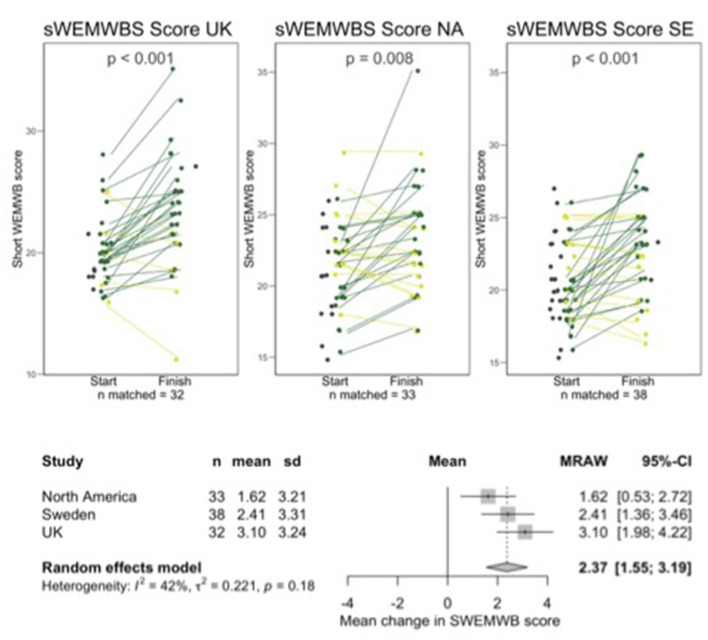
Line and forest plots for SWEMWBS. Dark green indicates improved scores, light green indicates worsening score or no change. Dark gray data points without a line represent people who started but did not finish or who completed a follow up questionnaire but could not be matched with a starting questionnaire.

**Figure 4:**
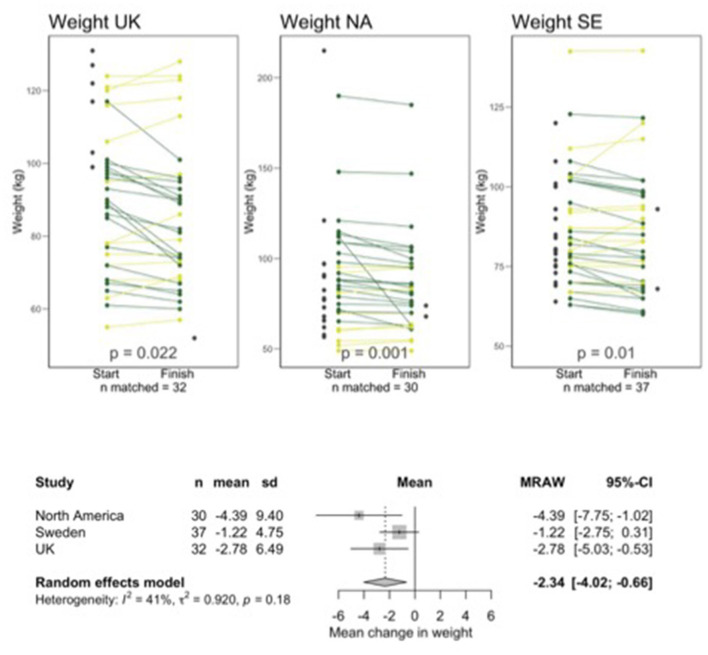
Line and forest plots for weight. Dark green indicates improved scores, light green indicates worsening score or no change. Dark gray data points without a line represent people who started but did not finish or who completed a follow up questionnaire but could not be matched with a starting questionnaire.

Figures 1–4 show line plots and forest plots for mYFAS2 score, CRAVED score, SWEMWBS score and weight. The line plot shows change over time for each participant across study locations. Improvement (e.g., decreased mYFAS2 score or increased SWEMWBS) is shown as dark green while the opposite change, or no change, is light green. Random effects forest plots calculate the overall change across all three settings.

All scores changed significantly from pre- to post-intervention. The mYFAS2 symptom score decreased, with a change of −1.52 (95% CI: −2.22, −0.81), CRAVED score decreased with a change of −1.53 (95% CI: −1.93, −1.13), SWEMWBS score increased with a change of 2.37 (95% CI: 1.55, 3.19) and weight decreased with a change of −2.34 kg (95% CI: −4.02, −0.66).

## Discussion

There is a dearth of published data on any intervention outcomes for individuals struggling with addictive behaviors relating to food. Meanwhile, clinicians and coaches are providing services to some clients seeking help. The data presented here represent an audit of three online low-carbohydrate “real food” programs with psychoeducation and social support currently delivered in three locations in North America and Europe. The vast majority of participants were female (91–100%), which is higher than is reflected in prevalence studies. Praxedes et al. (15) found 27% of males with food addiction in their review. However, there were only two studies found. Further studies are needed to establish suitable interventions for male individuals with food addiction.

The number of people requesting participation in the programs was notable, demonstrating that such programs are in demand. It was interesting that people inquiring about the programs were self-identifying as “food addicts” despite a lack of formal recognition of this condition in the health care system. As shown by the pre-program data, participants appear able to judge this well. There were no screening tools for EDs which would help separate the FA “signal” from the “noise” of dietary restraint (18). All 32 participants in the UK scored 3 or more on the 6 WHO criteria (CRAVED score) prior to the intervention, indicating a probable substance use disorder.

Retention at the end of the group sessions (~3 months) was 82.5, 48, and 70% for UK, NA, and SE, respectively. This is similar to other addiction programs such as those for smoking cessation where a meta-analysis showed an interquartile range of 68.5–89.5% for retention (36). NA retention is somewhat lower at this point. This difference cannot be attributed to larger group size as SE also ran larger groups. As more groups are audited further analysis of predictors of dropout such as higher weight or YFAS scores pre-program will be examined. Across all three countries participants independently set up support groups to share information between sessions, but no data were collected related to social support engagement. It appears that the interventions were accessible and acceptable to participants.

The significant improvements in FA symptoms across all three countries on both the mYFAS2 and CRAVED is encouraging, although this can be considered an early time point relative to the goal of evaluating outcomes after 2 years. Caution is required in interpretation of the results due to high relapse rates in any addictive disorder (37). Follow-up data will be published in due course. The mYFAS2 asks for symptoms during the last year but only 3 months had elapsed at follow-up, demonstrating that responses are influenced by current symptom experience.

Diets high in refined sugar and carbohydrate have been associated with poorer mental health (22). Gangwisch et al. (38) found that women with higher refined carbohydrates in their diet were more likely to have depression 3 years later. Current participants' mental wellbeing was lower than the reported UK norms for the SWEMWBS prior to the intervention (mean 23.5, SD 3.9) (26). However, post-intervention scores were similar to population norms. Improved wellbeing has a range of known beneficial effects on health and quality of life (39). Again, caution is required in interpreting these audit results and it will prove meaningful to ascertain whether these improvements are maintained at longer-term follow-up.

Weight loss is not always a key outcome of FA treatment because 11.4% of people with FA are of normal weight or underweight (40). Another study found 5.5% of normal weight and 15% of underweight people have food addiction (41). However, people often pursue treatment in the hope of achieving this goal, which is one reason that many ED professionals criticize this field (7). Weight loss was significant across the study sites at this stage of follow-up despite it not being a focus of the programs.

Individual variation in results from interventions is often lost in large data sets. The line plots in Figure 1 through Figure 4 show each participant's data which allow us to see the heterogeneity in responses. We hope to qualitatively explore factors contributing to variations in outcomes.

This audit has some limitations. There is no control arm to compare participants not receiving the intervention. Participants not completing the program and follow-up data may have had poorer outcomes than those completing the sessions (attrition bias). When more data are collected, it will be possible to qualitatively examine factors predicting drop out or poor results. Furthermore, the intensive contact with the clinicians and fellow participants can be therapeutic regardless of the nutrition intervention. The study did not include screening for eating disorders. It is known that FA and eating disorders often co-occur (15, 18, 20). It is possible that some of the variability in outcomes could be explained by taking this into account in future prospective studies.

## Conclusion

The current data are the first to demonstrate the short-term clinical effectiveness of a low-carbohydrate “real food” intervention delivered in an online group format with education and social support for individuals with FA symptoms. Larger, controlled and randomized intervention studies are urgently needed to continue to explore ways to help people with this serious and multi-faceted condition which often goes undiagnosed and untreated. It would be extremely useful to compare this approach to more inclusive “all foods fit” approaches among those with co-occurring FA and EDs, particularly BED.

## Data availability statement

The raw data supporting the conclusions of this article will be made available by the authors, without undue reservation.

## Ethics statement

Ethical review and approval was not required for the study on human participants in accordance with the local legislation and institutional requirements. The patients/participants provided their written informed consent to participate in this study.

## Author contributions

JU produced the first draft of the manuscript. JU, HG, CK, MP, FS, and CS commented on the manuscript, contributed equally to the protocols, clinical program, and data collection. CD analyzed the data sets, produced the statistics, and commented on the manuscript. DW was advisor to the project and contributed to and commented on the manuscript. All authors contributed to the article and approved the submitted version.

## Conflict of interest

Authors HG, CK, MP, FS, CS, and DW have fee paying clients with food addiction. Author DW was employed at Nutrition in Recovery LLC. The remaining authors declare that the research was conducted in the absence of any commercial or financial relationships that could be construed as a potential conflict of interest.

## Publisher's note

All claims expressed in this article are solely those of the authors and do not necessarily represent those of their affiliated organizations, or those of the publisher, the editors and the reviewers. Any product that may be evaluated in this article, or claim that may be made by its manufacturer, is not guaranteed or endorsed by the publisher.
