# Supplementary material for: Low carbohydrate and psychoeducational programs show promise for the treatment of ultra-processed food addiction
Source: Front Psychiatry. 2022 Sep 28;13:1005523. doi: 10.3389/fpsyt.2022.1005523 (PMC9554504; doi:10.3389/fpsyt.2022.1005523)
Supplement: Supplementary file 1 [file Data_Sheet_1.PDF]

An **ICD-10** based **food-behaviour** questionnaire

Name: Date:

Have you ever CRAVED certain foods or drinks?

This is a questionnaire to assess whether you experience cravings for foods or drinks that might make it difficult for you to sustain changes to your diet.

Spend a few minutes thinking about any foods or drinks you struggle to control your intake of. Include foods and drinks that occupy your mind. Write a list of these foods and drinks in the box:

**Now answer the following questions about these foods or drinks.**<br>**For each question, tick the relevant box on the right.**	**YES:** In the past month	**YES:** In the past year	**YES:** More than one year ago	**NO:** Never
1. Have you ever had such a strong desire or sense of compulsion at the thought of having these foods or drinks that you could not resist the urge to consume them?				
2. Have you ever noticed that you need to use increasing amounts of these foods or drinks to get the same effect compared to when you first had them?				
3. Have you ever noticed that you neglect planning activities because you are too tired, sick or preoccupied due to having too much of these foods or drinks?				
4. Have you ever consumed more of these foods or drinks than you intended on more than one occasion?				
5. Have you ever experienced at least two of the following withdrawal symptoms when you cut down or stopped consuming these foods or drinks?<br>• Headache<br>• Nausea or vomiting<br>• Anxiety<br>• Depressed or low mood<br>• Irritability<br>• Shakes<br>• Sweating<br>• Heart Racing /Palpitations<br>• Fast or shallow breathing<br>• Diarrhoea or constipation<br>• Sleep disturbance or vivid dreams				
6. Have you ever continued to consume these foods or drinks despite you or someone else believing that difficulties with weight gain, diabetes, memory, concentration, anxiety, unexplained mood swings, depression, panic attacks or other physical or mental health problems could be due to your consumption of these foods or drinks?				

Scoring: Three or more symptoms in the past year could indicate addictive behaviours related to certain foods and / or drinks.

In Development

For more information about the use and scoring of this tool, or to support the development & validation of its use:
Please contact: heidigiaever@yahoo.com and jenunwin@hotmail.co.uk
