# Supplementary material for: Low carbohydrate and psychoeducational programs show promise for the treatment of ultra-processed food addiction
Source: Front Psychiatry. 2022 Sep 28;13:1005523. doi: 10.3389/fpsyt.2022.1005523 (PMC9554504; doi:10.3389/fpsyt.2022.1005523)
Supplement: Supplementary file 2 [file Data_Sheet_2.PDF]

Fork in the Road Food Guide

EAT & ENJOY

Treat yourself to the best you can afford: organic, free-range, grass-fed.
You will also be saving money by not eating junk and take outs.

Beef, Lamb & Pork
Choose sausages with high meat content and no added sugar

Eggs, Chicken, Duck & Turkey

Fish & Shellfish
Including quality canned tuna

Game Meats
Bison, Goose, Elk, Rabbit, Pheasant & Ostrich

Offal
Bone Marrow, Kidney, Tongue, Liver & Heart

Lard & Tallow
Pork & Beef Fat

Butter & Ghee

Oils
Olive & Avocado Oil
Coconut Oil

Mustard & Mayonnaise
Avoid mayo made with vegetable oils.
Make your own.

Above-Ground Veggies
Cabbage, Cauliflower, Broccoli, Courgette/Zucchini, Onions, & Green Beans

Vegetable & Green Salads
Careful, dressings are often loaded with sugar

Avocados & Olives

Sauerkraut & Pickles
Check labels for sugar.
Make your own.

Herbs & Spices
Fresh Ginger & Garlic

Lemon & Lime

Carbonated & Still Water
Add a squeeze of lemon, lime or cucumber

Coffee & Tea
Coffee with no sugar or sweeteners
Herb & Fruit Tea, Black Tea
Ideally decaffeinated

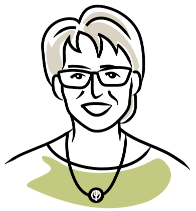

"A low-carb way of eating is not a deprivation diet, but rather a celebration of a life eating delicious food."

DR JEN UNWIN

VENTURE WITH CAUTION

Add to the abstain list if you tend to overeat these

Starchy Vegetables
Potatoes, Peas, Sweetcorn, Sweet Potato, Beetroot & Carrot

Legumes & Beans
Can cause bloating for some folks.

Nightshades
Tomatoes, Eggplant & Peppers

Apples, Peaches, Melons, Pears & Berries

Bacon, Salami & Cured Meats
Check labels for sugar

Almond & Coconut Flour

90% Dark Chocolate

Nuts & Seeds
Especially salted

Nut Butters
Peanut, Almond & Cashew

Full-Fat Greek Yoghurt

Cream & Milk
Cream has less sugar than milk in coffee.
Dairy is problematic for some people.
Goat and sheep milk can be better.

Cheese

ABSTAIN & REFRAIN

This can seem daunting at first, but becomes normal with time

Refined Table Sugar

Natural Sugars
Honey & Maple Syrup

Sweeteners
Alcohol & Artificial

Vegetable & Seed Oils
Margarine, Canola (Rapeseed) Oil, Sunflower & Safflower Oil

Alcohol

Sugary Drinks
Soda Pop, Apple & Orange Juice

Fake Milks
Soy, Rice & Oat Milk

Grains & Starches
Bread, Rice, Potatoes, Pasta, Crisps & Chips

Biscuits/Cookies
Pastry, Cakes & Candy

Smoothies, Ice Cream
& Low-Fat Yoghurt

Tropical Fruit
Bananas, Pineapple & Mangoes

Dried Fruit,
Raisins & Grapes

Ketchup
Condiments with Sugar
