# Supplementary material for: Low carbohydrate and psychoeducational programs show promise for the treatment of ultra-processed food addiction
Source: Front Psychiatry. 2022 Sep 28;13:1005523. doi: 10.3389/fpsyt.2022.1005523 (PMC9554504; doi:10.3389/fpsyt.2022.1005523)
Supplement: Supplementary file 3 [file Presentation_1.PPTX]

## Slide 1
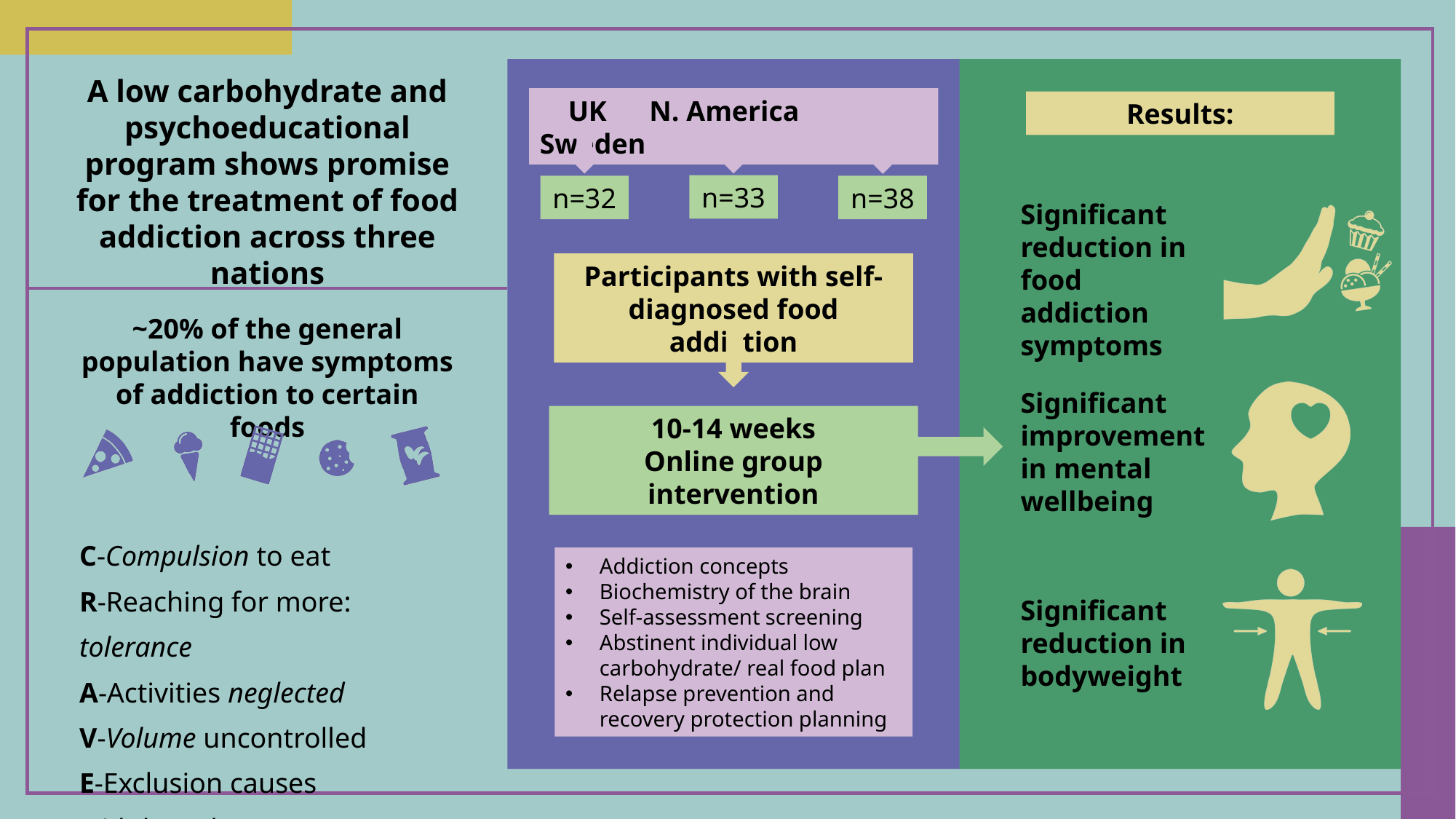

Pizza, chocolate, crisps, cookies, ice cream…
A low carbohydrate and psychoeducational program shows promise for the treatment of food addiction across three nations
 UK N. America Sweden
Results:
#
n=33
n=32
n=38
Significant reduction in food addiction symptoms
Participants with self-diagnosed food addiction
~20% of the general population have symptoms of addiction to certain foods
C-Compulsion to eat
R-Reaching for more: tolerance
A-Activities neglected
V-Volume uncontrolled
E-Exclusion causes withdrawal
D-Despite damage, can’t stop
Significant improvement
in mental
wellbeing
10-14 weeks
Online group intervention
Addiction concepts
Biochemistry of the brain
Self-assessment screening
Abstinent individual low carbohydrate/ real food plan
Relapse prevention and recovery protection planning
Significant reduction in bodyweight
