# Supplementary material for: Low carbohydrate and psychoeducational programs show promise for the treatment of ultra-processed food addiction
Source: Front Psychiatry. 2022 Sep 28;13:1005523. doi: 10.3389/fpsyt.2022.1005523 (PMC9554504; doi:10.3389/fpsyt.2022.1005523)
Supplement: Supplementary file 4 [file Table_1.DOCX]

**Supplement A**

**Glossary**

**BED Binge eating disorder**

**BMI Body mass index**

**BN Bulimia nervosa**

**CRAVED Questionnaire for FA based on ICD-10 SUD criteria**

**DSM-5 Diagnostic and statistical manual of mental disorders**

**ED Eating disorder**

**FA Food addiction**

**ICD-10 International classification of diseases, WHO**

**NA North America**

**SE Sweden**

**SUD Substance use disorder**

**YFAS Yale food addiction scale**

**mYFAS2 Modified Yale food addiction scale version 2**

**SWEMWBS Warwick-Edinburgh mental wellbeing scale, short version**

**UK United Kingdom**

**WHO World Health Organisation**
