# Supplementary material for: Low carbohydrate and psychoeducational programs show promise for the treatment of ultra-processed food addiction
Source: Front Psychiatry. 2022 Sep 28;13:1005523. doi: 10.3389/fpsyt.2022.1005523 (PMC9554504; doi:10.3389/fpsyt.2022.1005523)
Supplement: Supplementary file 5 [file Table_2.DOCX]

**Supplement B**

**Information Sheet: Food Addiction Recovery**

Thank you for your interest in joining the Food Addiction Recovery programme.

Heidi Giaever (nutrition consultant) and Jen Unwin (clinical psychologist) are running a group programme and follow-up, based on the best international current evidence and practice for food addiction recovery.

The initial intensive programme consists of 10 weekly, 90 minute virtual group meetings with 8-15 people. Everyone joining has self-identified as having a food/sugar addiction problem and has spoken to either Heidi or Jen. Follow-up is monthly support meetings via zoom.

Over the 10 weeks we will cover the following:

- food addiction concepts.

- understanding the extent to which you have been 'embedding' your addiction.

- understanding how different foods build or destroy our bodies and brains.

- understanding how to create and develop your own tool-box with practical tools, and practice using them, to help you free yourself from the controlling influence that certain foods have over you.

We will be auditing the outcomes carefully by asking you to complete an anonymised online survey before, after, at 6 months, 12 months, 18 months and 2 years post the group sessions. The survey will take around 10-15 minutes each time and will ask about your current relationship with food and your mental wellbeing. We hope to publish the findings of the audit, using anonymous data analysed by an independent statistician.

The study is part of a three-centre project. The other teams are in Sweden and North America and will be running similar groups with follow up and administering the same survey. There is very little research on food addiction treatment, so the audit will make a significant contribution to the literature and to helping others in the future.

We don’t foresee any risks to you taking part, but it is **important that you discuss any dietary changes with your healthcare team** **if you are taking medication**.

If you are happy to take part and consent to the use of your anonymised data for publication of the findings, we will send you the first survey to complete which includes you giving your informed consent.

If you have any questions at all, you can email Heidi Giaever [heidigiaever@yahoo.com](mailto:heidigiaever@yahoo.com) or Jen Unwin [jenunwin@hotmail.co.uk](mailto:jenunwin@hotmail.co.uk) . This research is being conducted with the support of the Public Health Collaboration [www.phcuk.org](http://www.phcuk.org) and you can contact the trustees there if you have any concerns or complaints about the project that you feel have not be answered by Heidi or Jen. <https://phcuk.org/contact/>
