# Supplementary material for: Low carbohydrate and psychoeducational programs show promise for the treatment of ultra-processed food addiction
Source: Front Psychiatry. 2022 Sep 28;13:1005523. doi: 10.3389/fpsyt.2022.1005523 (PMC9554504; doi:10.3389/fpsyt.2022.1005523)
Supplement: Supplementary file 6 [file Table_3.DOCX]

**Supplement C**

Recruitment Protocol

We are looking for self-referred and self-diagnosed food addicts who identify with the following self-assessment:

- I am addicted to sugar, sweets, carbs, snacking, processed foods, industrial foods such as pizza, chocolate, crisps (chips), biscuits (cookies), ice cream, chips (fries), burgers, fizzy drinks (pop), cake, cereals, breads etc.
- I am a life-long ‘chocoholic’ or ‘carboholic’ and it is harming me and my health
- I have no control over certain foods
- I can’t stop myself overeating cert ain foods
- I know what to eat but can’t stick to it
- I have tried many diets but can’t ‘stay stopped’ with certain foods

Exclusion Criteria

- Under 18 years of age
- Pregnant
- Serious mental health problem and consequently clinician recommends against
- Any exclusion objection from the person’s healthcare provider

Participant screening interview

Each prospective participant was interviewed by one of the clinicians in their corresponding team location

- Do you feel you have some form of food or sugar addiction that you need help to deal with?
- Are you joining this online group program for your own benefit, not having been persuaded against your better judgement by anyone?
- Do you feel able to participate in group sessions with people with similar challenges but who may have different experiences?
- Have you received, read and understood the details in the information sheet about the program and anonymous data collection?
- Can you confirm that you have no other physical health or mental health challenges which mean you should not take part
- What current medications are you taking? Patients taking diabetes or blood pressure medications will be advised to discuss participation and medication management with their healthcare provider prior to the start of the program
