# Supplementary material for: Low carbohydrate and psychoeducational programs show promise for the treatment of ultra-processed food addiction
Source: Front Psychiatry. 2022 Sep 28;13:1005523. doi: 10.3389/fpsyt.2022.1005523 (PMC9554504; doi:10.3389/fpsyt.2022.1005523)
Supplement: Supplementary file 7 [file Table_4.DOCX]

Supplement E

Program content comparison UK, NA, SE

| Session | UK | NA | SE |
| --- | --- | --- | --- |
| 1 | Addiction concepts | Introduction to food addiction & mindful eating | Mapping consequences |
| 2 | Self-assessment screening | Abstinence & withdrawal/food plan | Addiction screening |
| 3 | Biochemistry/food plan | Addiction interaction | Motivation and guiding light |
| 4 | Sugar as a drug | Craving management/cues | The food |
| 5 | Best hopes | Building hope | My triggers |
| 6 | Resources | Thought and feelings, managing slips | Food planning |
| 7 | Habits & Tastes | Emotional management, movement and spirituality | Relapse prevention |
| 8 | Eating to live | Stress management | Stress factors |
| 9 | Relapse prevention | Body image | Biochemical repair |
| 10 | Recovery protection | Self-esteem and compassion | Digestive system |
| 11 |  |  | Candida |
| 12 |  |  | Thoughts and emotions |
| 13 |  |  | Power of habits |
| 14 |  |  | Follow up |
